# Influence of Mixing Duration and Absorption Characteristics of Superabsorbent Polymers on the Fresh and Hardened Properties of High-Performance Concrete

**DOI:** 10.3390/ma18153609

**Published:** 2025-07-31

**Authors:** Yu-Cun Gu, Kamal H. Khayat

**Affiliations:** Department of Civil, Architectural, and Environmental Engineering, Missouri University of Science and Technology, Rolla, MO 65409, USA; gywvy@mst.edu

**Keywords:** superabsorbent polymer, absorption kinetics, mixing sequence, rheology, internal curing, high-performance concrete

## Abstract

This study investigates the combined influence of superabsorbent polymers (SAPs) with distinct absorption kinetics and extended mixing sequences on the rheological, mechanical, and transport properties of high-performance concrete (HPC). Two SAPs—an ionic acrylamide-co-acrylic acid copolymer (SAP-P) and a non-ionic acrylamide polymer (SAP-B)—were incorporated at an internal curing level of 100%. The impact of extended mixing times (3, 5, and 7 min) following SAP addition was systematically evaluated. Results showed that longer mixing durations led to increased superplasticizer demand and higher plastic viscosity due to continued water absorption by SAPs. However, yield stress remained relatively stable owing to the dispersing effect of the added superplasticizer. Both SAPs significantly enhanced the static yield stress and improved fresh stability, as evidenced by reduced surface settlement. Despite the rheological changes, mechanical properties—including compressive and flexural strengths and modulus of elasticity—were consistently improved, regardless of mixing duration. SAP incorporation also led to notable reductions in autogenous and drying shrinkage, as well as enhanced electrical resistivity, indicating better durability performance. These findings suggest that a 3 min extended mixing time is sufficient for effective SAP dispersion without compromising performance.

## 1. Introduction

Superabsorbent polymers (SAPs) are cross-linked polyelectrolytes that function as internal curing (IC) agents in cement-based materials and can be classified as either ionic (e.g., acrylamide-co-acrylic acid copolymers, acrylic polymers) or non-ionic (e.g., acrylamide polymers) [1,2]. Their efficacy in mitigating autogenous shrinkage and enhancing performance in high-performance concretes (HPCs) and ultra-high-performance concretes (UHPCs) depends on several parameters, including dosage [3,4], molecular structure [5,6], and particle size [7,8]. Incorporation of SAPs in dry form—rather than pre-saturated—has been shown to offer advantages such as improved dispersion and ease of handling during mixing [9,10].

Numerous studies have examined the influence of SAPs on the rheological properties of cementitious systems, particularly under conditions where additional water is introduced to compensate for SAP absorption and maintain fluidity [1,11,12,13,14,15,16]. For example, the use of acrylamide-co-acrylic acid copolymers (poly-AA-co-AM) has been reported to increase yield stress by approximately 100–400% [11]. Additionally, SAPs with larger particle sizes, such as poly-AA-co-AM and acrylic polymers (poly-AA), demonstrated higher absorption rates and longer absorption durations, resulting in significant increases in both yield stress (50–100%) and plastic viscosity (20–100%) over time in UHPC mixtures [12]. Non-ionic acrylamide polymers (poly-AM) have similarly been shown to elevate yield stress due to their prolonged absorption kinetics [1]. These findings collectively highlight that the development of rheological properties in SAP-modified mixtures is intricately governed by the time-dependent absorption and desorption behavior of the SAPs.

Despite these advances, the impact of mixing sequence—particularly extended mixing time—on SAP behavior has not been thoroughly studied. As absorption kinetics and dispersion of SAPs are sensitive to mixing conditions, it is critical to assess how these factors influence fresh concrete properties, especially in high-performance systems where workability and rheological control are essential.

Beyond rheology, SAPs are widely recognized for their role in mitigating autogenous shrinkage in concrete with low water-to-binder (w/b) ratios. By slowing the rate of internal moisture loss, SAPs reduce the development of early-age shrinkage and delay the onset of microcracking. Their effectiveness depends on various factors, including SAP dosage [3,7,17,18], type [3,18], particle size [7,17], and pretreatment [19] of SAP, and w/b [20,21]. Additionally, SAPs have gained attention for their role in enhancing self-healing and self-sealing capabilities in concrete [22]. As internal water reservoirs, SAPs can absorb moisture from humid environments and release it under drying conditions, thereby promoting continued hydration and aiding in crack healing. Studies have shown that SAPs can facilitate the closure of microcracks through binder hydration [23], and when used in conjunction with other healing agents such as calcium carbonate (CaCO_3_), they can further enhance healing efficiency [24]. Snoeck et al. [24] demonstrated that mortars reinforced with microfibers and subjected to wet–dry cycles exhibited substantial CaCO_3_ precipitation within cracks, enabled by SAP-assisted moisture retention.

While prior research has extensively addressed the effects of SAPs on shrinkage mitigation, permeability, and mechanical performance, few studies have systematically examined how extended mixing time influences SAP dispersion and performance, particularly in HPC mixtures.

The objective of this study is to comprehensively investigate the effects of SAP absorption behavior and mixing sequence on the fresh and hardened properties of HPC. Specifically, the study explores the influence of extended mixing times (3, 5, and 7 min) on workability, rheology (including yield stress, plastic viscosity, and static yield stress), and mechanical properties (compressive strength, flexural strength, and modulus of elasticity). Furthermore, the study evaluates how the interaction between SAP characteristics and mixing duration affects transport-related properties, providing a holistic assessment of SAP performance in HPC systems.

## 2. Materials and Methods

### 2.1. Materials

**Figure 1:**
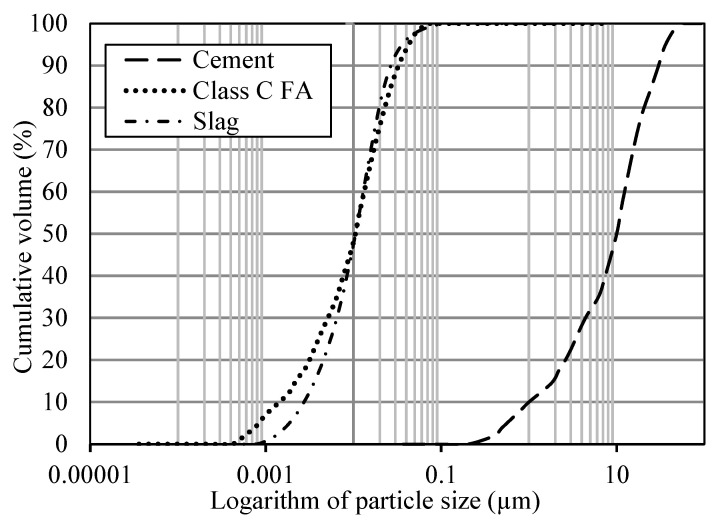
Particle size distribution of cementitious materials.

**Figure 2:**
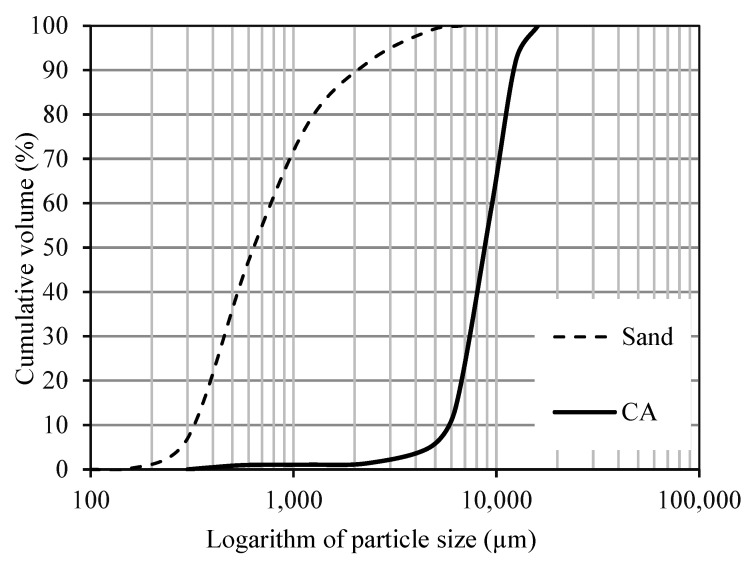
Grain size distributions of fine and coarse aggregates.

A Type I ordinary Portland cement (OPC) conforming to ASTM C150 [25], Class C fly ash (FA) in accordance with ASTM C618 [26], and ground-granulated blast-furnace slag (slag) meeting ASTM C989 [27] were used as the primary cementitious materials. Their physical and chemical properties, including Blaine fineness, are summarized in Table 1, with chemical compositions determined by X-ray fluorescence (XRF). The particle size distributions of OPC, FA, and slag are shown in Figure 1. River sand and locally sourced crushed limestone from Missouri served as fine and coarse aggregates, respectively. The fine aggregate had a fineness modulus of 2.6 and specific gravity of 2.57, while the coarse aggregate had a nominal maximum size of 25 mm, specific gravity of 2.72, and SSD absorption of 1%. Particle size distributions for both aggregates are presented in Figure 2.

A polycarboxylate-based superplasticizer (SP) and a synthetic resin-type air-entraining agent (AEA) were used to achieve the desired workability and air content. An initial slump of 215 ± 15 mm and an air content of 5–6% are specified to ensure adequate workability for placement and finishing, while maintaining the required durability of the pavement in freeze–thaw conditions, in accordance with Illinois Tollway standards. A hydration retarder composed of sucrose, gluconates, phosphates, and lignosulfonates was employed to regulate the setting behavior of the cementitious system. All chemical admixtures used in this study met the requirements of ASTM C494 [28].

Two types of SAPs with distinct molecular structures and particle sizes were used in this study. The first, SAP-P, is an angular, covalently cross-linked acrylamide-co-acrylic acid polymer (poly-AA-co-AM), while the second, SAP-B, is a non-ionic acrylamide-based polymer (poly-AM) with a similar angular morphology. The mean particle sizes of SAP-P and SAP-B were 850 μm and 63 μm, respectively.

### 2.2. Experimental Program

#### 2.2.1. Absorption Capacity Measurement

**Figure 3:**
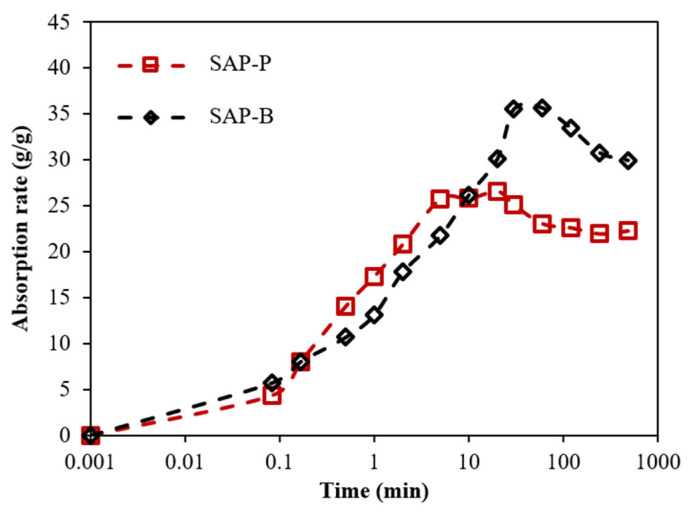
Absorption capacity of investigated SAPs over time in filtrated solution.

The absorption capacity of the SAPs was determined using the tea bag method [29] in a filtrated pore solution, both with and without chemical admixtures. The solution was derived from a ternary binder composition comprising 52% OPC, 35% slag, and 13% FA, and extracted from a paste mixture with a w/b of 5. To evaluate absorption kinetics, approximately 0.2 g of dry SAP was placed into a pre-weighed tea bag. The sealed tea bag was then submerged in 500 mL of the test solution. At specified time intervals—10 s, 20 s, 30 s, 1 min, 5 min, 10 min, 30 min, 60 min, 2 h, 4 h, and 8 h—the tea bag was removed, placed on a dry cloth, gently blotted with another cloth for approximately 10 s to remove excess and weakly bound liquid, and then weighed. The absorption capacity was calculated based on the weight gain of the tea bag over time. The times for SAP-P and SAP-B to reach maximum liquid absorption were 10 and 60 min, respectively, as shown in Figure 3.

#### 2.2.2. Mixture Proportion and Mixing Procedure

The mixture proportions of the investigated HPC mixtures are provided in Table 2. A ternary binder system was used, consisting of 35% slag and 13% FA as volume replacements for OPC. To ensure effective IC, the dosage of SAP was selected such that the majority of the paste volume remained within the effective travel distance of the curing water, as recommended in previous studies [11]. Accordingly, two SAP dosages corresponding to 50% and 100% IC levels were evaluated. The required mass of SAP to compensate for chemical shrinkage was calculated in accordance with ASTM C1761/C1761M [30], using the following equation:

M_sap_ = (C_f_ × C_S_ × α_max_)/(S × W_sap_) (1)

where C_f_ is the binder content (kg/m^3^); C_S_ is the chemical shrinkage of the binder, 0.07 cm^3^/g; α_max_ is the maximum potential degree of hydration of the binder, 0.91 used in this study; S is the absorption rate of SAP in the filtrated pore solution; and W_sap_ is the desorption rate of the SAP upon equilibrium at a relative humidity of 94% expressed as a fraction of oven-dry mass.

All mixtures were prepared with a w/b of 0.37. The base SP dosage was fixed at 580 mL/m^3^, with additional SP added as needed to compensate for the loss of free water due to SAP absorption, to maintain a target initial slump of 215 ± 15 mm. The AEA dosage was adjusted to achieve an air content of 5–6%, and a set retarder was incorporated to mitigate slump loss associated with SAP absorption.

All concrete mixtures were prepared using a 120 L drum mixer (Crown Concrete Mixer Machine) under ambient laboratory conditions. The batching and mixing sequence proceeded as follows: coarse aggregate and sand were first blended with half of the total mixing water—pre-diluted with the air-entraining agent (AEA)—for 1 min. Subsequently, the binder components (cement, fly ash, and slag) were introduced along with one-quarter of the mixing water and the set retarder, followed by mixing for 30 s. The remaining quarter of the mixing water, along with the initial dosage of superplasticizer (SP), were then added, and mixing continued for an additional 90 s.

Initial fresh properties—slump, air content, and unit weight—were measured within 5 min of completing the first mixing stage. The mixture was then remixed for 2 min, during which the superabsorbent polymer (SAP) was added using a funnel to ensure even dispersion. This was followed by an extended mixing phase of either 3, 5, or 7 min, depending on the test condition. Additional SP was introduced during this stage as needed to compensate for water absorbed by the SAP and to maintain the target slump of 215 ± 15 mm. Fresh properties were again measured at the end of the second mixing stage.

To assess workability retention, slump was remeasured at two subsequent time intervals—35 min and 55 min after the initial contact between cement and water. During these two 20 min intervals, the concrete was kept at rest for 4 min and agitated for 1 min at 5 min intervals using high-speed mixing.

#### 2.2.3. Fresh Properties Measurement

**Figure 4:**
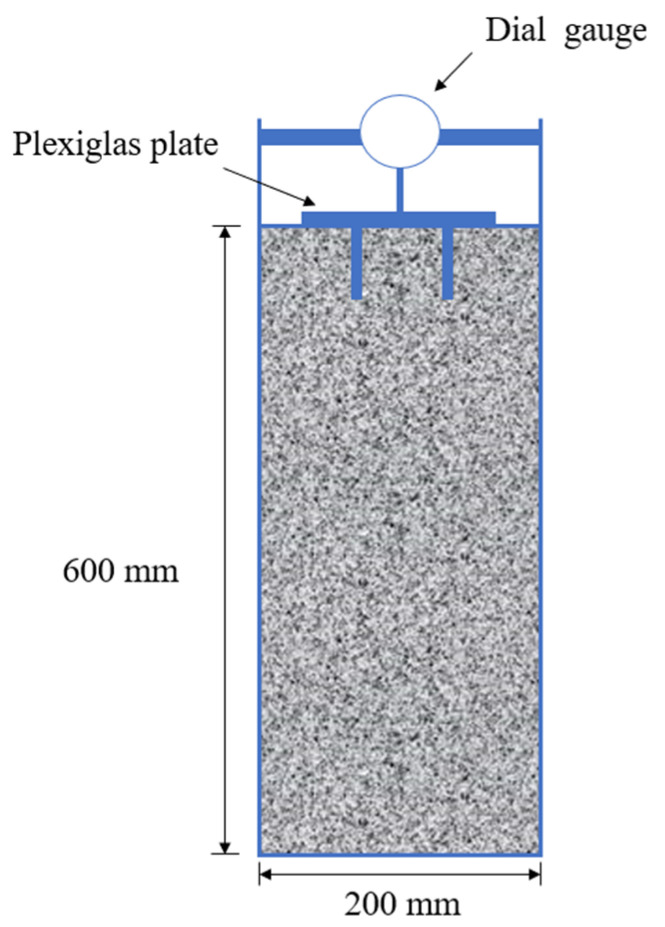
Schematic of the concrete surface settlement test.

The fresh properties of the concrete mixtures—including slump, unit weight, air content, and surface settlement—were evaluated. Slump and air content were measured approximately 5 min after water–binder contact for the reference mixture, and at approximately 15 min for the SAP-modified mixtures. Unit weight and air content were determined in accordance with ASTM C138 [31] and ASTM C231 [32], respectively, while slump was measured according to ASTM C143 [33]. Mixture stability was assessed using a surface settlement test [34], as illustrated in Figure 4. Fresh concrete was placed in a PVC column with a diameter of 200 mm and a height of 600 mm. A 0.002 mm dial gauge, fixed to an acrylic plate resting on the concrete surface, was used to monitor settlement over time until a steady-state condition was reached. The final settlement was expressed as a percentage by dividing the measured displacement by the column height (600 mm). All tests were conducted in duplicate to verify result reproducibility.

#### 2.2.4. Rheology Measurements

Rheological properties were measured at 10 min for the reference mixture and at 20 min for the SAP-modified mixtures, relative to the time of initial water–binder contact. To minimize the influence of structural build-up during the 5 min interval required for fresh property measurements, the concrete was thoroughly remixed prior to rheological testing. The yield stress and plastic viscosity were determined using a ConTec 5 rheometer. Prior to testing, the fresh concrete was pre-sheared at a constant rotational speed of 0.4 revolutions per second (rps) for 30 s. The rotational speed then gradually decreased from 0.4 to 0.025 rps in 10 incremental steps. At each step, 50 torque measurements were recorded and analyzed for flow stability, plug flow behavior, and potential segregation, following the established procedures [35]. Only steady-state torque values were used to compute the yield stress and plastic viscosity using the Bingham model. The Reiner–Riwlin transformation equations were applied to convert torque and rotational speed data into rheological parameters.

#### 2.2.5. Mechanical Properties Measurements

Compressive strength was evaluated at 7, 14, 28, and 56 days using 100 × 200 mm cylindrical specimens, in accordance with ASTM C39 [36]. Specimens were capped with a high-strength sulfur compound in compliance with ASTM C617 [37]. The loading rate was controlled to achieve a compressive stress increase of 0.25 ± 0.05 MPa/s. MOE was measured using the same specimen dimensions according to ASTM C469 [38]. Testing involved three loading cycles, with the testing frame’s moving head set to a displacement rate of approximately 1.27 mm/min. Each specimen was loaded until the applied force reached 40% of the average ultimate load obtained from companion specimens. Flexural strength was assessed using prismatic beams measuring 75 × 75 × 400 mm with a span length of 300 mm, tested according to ASTM C1609 [39]. Displacement control was maintained at a rate of 0.088 mm/min until specimen failure. The flexural strength was calculated using the following equation:

F = PL/bd^2^ (2)

where F is the strength (MPa); P is the maximum load (N); L is the span (mm); b is the average width of the sample (mm); and d is the average depth of the sample (mm).

#### 2.2.6. Drying Shrinkage

Drying shrinkage was measured in accordance with ASTM C157 [40] using prismatic specimens with dimensions of 75 × 75 × 280 mm. A digital-type extensometer was used to record length changes. Following demolding, the specimens were immersed in lime-saturated water for 1 h, after which the first reading was taken. The specimens were then cured in lime-saturated water for an additional 6 days. The length measured at the end of the 7-day curing period was designated as the initial reference length for shrinkage calculations.

Autogenous shrinkage was evaluated according to ASTM C1698 [41], which was conducted using samples cast in standard corrugated plastic tubes. Each tube measured 420 mm in length, with a diameter of 30 mm at the ridges and 25 mm at the grooves. The testing procedure followed the typical practice for monitoring volume changes under sealed conditions. Concrete-equivalent mortar was used in this study to measure the autogenous shrinkage. Shrinkage measurements commenced approximately 18 h after initial water–cement contact, with the final setting time defined as the zero reference point. Subsequent measurements were taken at 6 and 12 h after the final set, followed by daily readings during the first week and weekly intervals thereafter until 28 days.

#### 2.2.7. Transport Properties Measurement

Electrical resistivity measurements were conducted to assess the corrosion resistance classification of concrete. Tests were performed on saw-cut cylindrical specimens at 28 days of age. Two measurement methods were employed: the direct two-electrode method, in accordance with ASTM C1760 [42], which determines bulk electrical conductivity; and the four-point Wenner probe method, following AASHTO TP 95-11 [43], which measures surface resistivity. These methods collectively provide a comprehensive evaluation of the concrete’s electrical performance related to its potential corrosion behavior.

## 3. Results and Discussion

### 3.1. Fresh Properties

#### 3.1.1. Coupled Effect of SP Demand, SAP Absorption, and Mixing Time on Workability

Table 3 presents the fresh properties of the investigated HPC mixtures incorporating 100% IC using SAP-P and SAP-B, with extended mixing times of 3, 5, and 7 min following SAP addition. The evaluated properties include initial slump, air content, and unit weight. Results indicate that a longer mixing duration after SAP addition required a higher dosage of SP to maintain the target initial slump of 215 ± 15 mm and slump flow of 400–500 mm. For example, the mixture containing 100% IC with SAP-P and a 3 min extended mixing time required a 60% higher SP dosage, while the mixture with a 7 min extended mixing time required a 127% increase, both relative to the reference mixture. This increase in SP demand with prolonged mixing time is attributed to the continued moisture absorption by SAP particles during mixing. Both SAP-P and SAP-B continued to absorb water from the surrounding filtrate solution for up to 30 min.

Similarly, the sustained water uptake of SAP-P and SAP-B within the first 30 min led to more pronounced slump loss. For example, a mixture containing SAP-P showed a slump reduction of about 150 mm after 40 min of extended mixing. Extended mixing also slightly elevated the air content; in the case of SAP-B, increasing the mixing time from 3 to 7 min raised the air content by approximately 0.5%. During the testing of slump retention and rheological parameters over time, the materials were agitated for 1 min every 5 min; this extended agitation led to a slight increase in air content.

#### 3.1.2. Influence of SAP on the Stability of Fresh HPC

**Figure 5:**
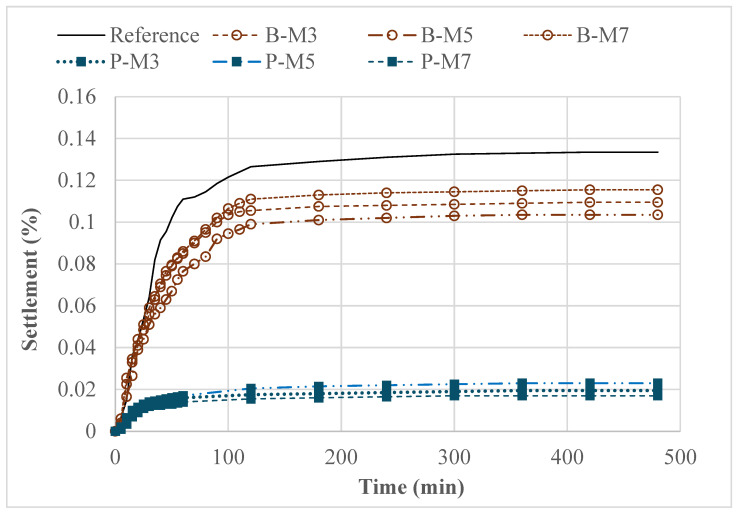
Surface settlement of HPC made with SAPs and different extended mixing times.

Figure 5 illustrates the surface settlement (%) of the investigated HPC mixtures incorporating SAPs under varying extended mixing times following SAP addition. The results indicate a substantial reduction in surface settlement with the inclusion of SAPs, which is advantageous for minimizing formwork pressure and facilitating concrete placement in challenging conditions, such as on inclined surfaces. Notably, the use of SAP-P led to a marked decrease in surface settlement, from 0.135% to 0.02%. This improvement is primarily attributed to the significant increase in plastic viscosity induced by SAP absorption, as further elaborated in the subsequent section.

Moreover, extended mixing time exhibited minimal influence on the surface settlement of mixtures containing SAPs. For example, mixtures incorporating 100% IC with SAP-B and extended mixing times from 3 to 7 min exhibited surface settlement values ranging narrowly from 0.10% to 0.12%. Overall, the observed reduction in surface settlement due to SAP incorporation highlights their efficacy in enhancing the stability of fresh HPC mixtures. This effect is particularly relevant in applications where dimensional accuracy and surface integrity are critical, such as in precast elements or concrete cast on inclined planes. This stability simplifies process control during mixing and offers practical benefits in field applications where precise timing may be difficult to maintain.

### 3.2. Rheological Properties

Table 4 presents the yield stress and plastic viscosity of HPC mixtures incorporating two types of SAPs subjected to varying extended mixing durations. The results reveal that plastic viscosity increased progressively with longer mixing times for both SAP types, whereas yield stress remained largely unaffected. For example, the mixture containing SAP-P exhibited a 64% increase in plastic viscosity at a 7 min mixing duration compared to its 3 min counterpart. Such an increase can result in a significant improvement in the stability of fresh concrete, as illustrated in Figure 5. This trend can be attributed to the ongoing moisture absorption by the SAPs during prolonged mixing, which reduces the volume of free water and enhances the mixture’s resistance to flow. Conversely, the required increase in SP dosage at longer mixing durations counteracted the viscosity-induced rise in yield stress. As a result, the yield stress remained relatively stable across the mixing times, indicating a complex interplay between SAP absorption and SP dispersing effects. In addition, the incorporation of both SAP types led to an increase in static yield stress, measured after a 5 min rest period. For example, SAP-B increased the 5 min static yield stress by approximately 90–110% across all extended mixing times. SAP-P also significantly enhanced static yield stress, with increases of 157% and 168% observed at 3 and 5 min of extended mixing times, respectively. However, at a 7 min mixing time, the increase was notably reduced to only 30%. These findings are consistent with those reported by Gu et al. [44,45], who investigated the influence of SAP absorption kinetics on the structural buildup of 3D printable concrete. Their study revealed that static yield stress development is strongly influenced by the extent of moisture absorption occurring within the rest period. In the present study, the limited increase observed for SAP-P at 7 min is attributed to the SAP reaching absorption equilibrium after approximately 10 min in the filtrate solution, which coincides with the end of mixing. Consequently, minimal additional water uptake occurred during the rest period, resulting in a lower structural buildup. Overall, the use of SAP and additional superplasticizer, combined with different extended mixing times, resulted in an increased yield stress, which reduced formwork pressure and improved stability without compromising the suitability of the concrete for pavement construction.

### 3.3. Mechanical Properties

**Figure 6:**
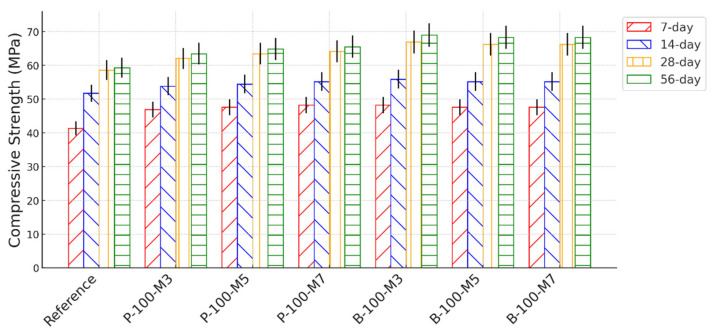
Compressive strength of HPC made with SAPs and different extended mixing times.

Figure 6 presents the compressive strength of the HPC mixtures incorporating SAPs, prepared with varying extended mixing times (i.e., 3 min, 5 min, and 7 min) and tested at different curing ages. The results indicate that the use of SAP-P and SAP-B enhanced the compressive strength by approximately 10–15% and 10–20%, respectively. These findings are consistent with previous studies, which have shown that internal curing (IC) provided by SAPs can improve compressive strength by promoting continued hydration. However, it is also recognized that the desorption of SAPs can leave behind voids that partially offset the beneficial effects of IC. This dual effect was also observed in the present study. Furthermore, variations in extended mixing times exhibited minimal influence on compressive strength. For example, the mixtures prepared with SAP-P showed compressive strength deviations within 5% across all mixing durations. This suggests that a 3 min extended mixing time was sufficient to achieve uniform dispersion of both SAP types, ensuring consistent mechanical performance.

**Figure 7:**
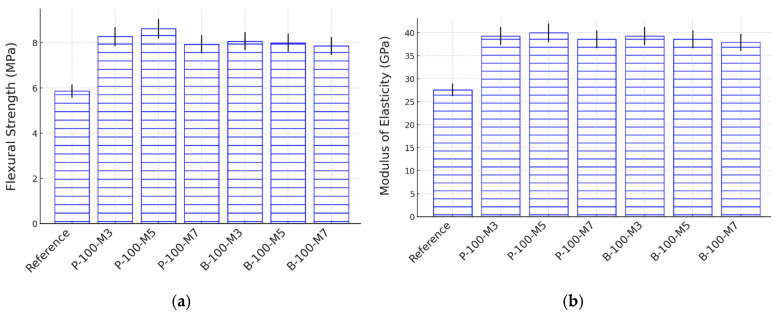
Flexural strength (**a**) and MOE (**b**) of HPC made with SAPs and different extended mixing times at 28 days of age.

Figure 7a,b present the 28-day flexural strength and MOE of HPC mixtures incorporating SAPs, prepared with varying extended mixing times (i.e., 3 min, 5 min, and 7 min). The incorporation of SAPs resulted in a 5–15% increase in flexural strength, which is similar to the improvements observed in compressive strength. More notably, the 28-day MOE exhibited a substantial enhancement of 15–25% with SAP addition. For instance, the use of SAP-P with a 3 min extended mixing time increased the MOE by 25% compared to the reference mixture.

Moreover, the variation in extended mixing time had no significant effect on either flexural strength or MOE at 28 days. All mixtures containing SAPs exhibited consistent improvements in these mechanical properties across the three mixing durations. For example, the flexural strength of mixtures with SAP-P increased by 14–17% regardless of the extended mixing time. These findings suggest that a 3 min extended mixing time is adequate to achieve uniform SAP dispersion, thereby maximizing their mechanical benefits without requiring prolonged mixing.

### 3.4. Viscoelastic Properties

**Figure 8:**
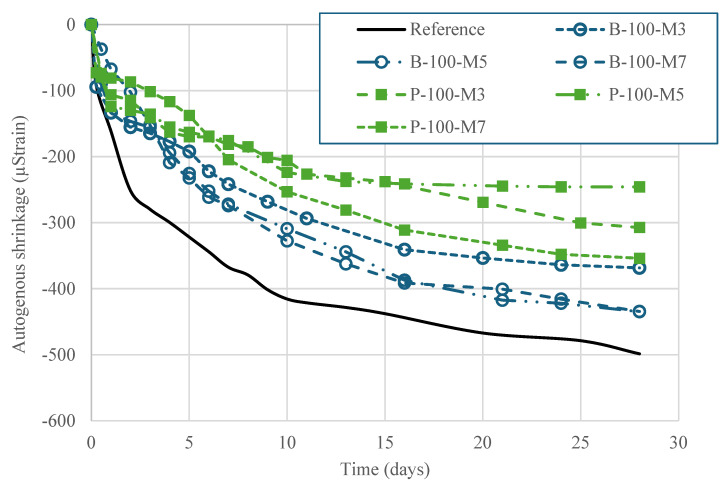
Autogenous shrinkage of HPC made with SAPs and different extended mixing times.

**Figure 9:**
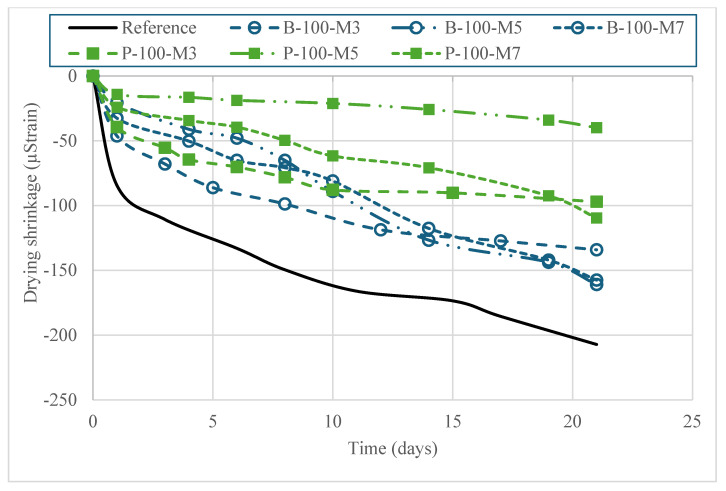
Drying shrinkage of HPC made with SAPs and different extended mixing times (initial length was recorded at 7 days of age after air drying).

Figure 8 illustrates the autogenous shrinkage behavior of the HPC mixtures incorporating SAPs, prepared with three different extended mixing times (3, 5, and 7 min). The results clearly indicate that the inclusion of SAPs markedly reduced autogenous shrinkage in comparison to the reference mixture without SAP. Notably, the 28-day autogenous shrinkage of the mixtures incorporating SAP-P was reduced by approximately 30–50% across all mixing durations. Internal curing by SAPs improves concrete performance through two mechanisms: (i) supplying additional water to compensate for shrinkage, and (ii) homogenizing moisture distribution and regulating water desorption over time. In this study, with a moderate w/b of 0.37 and no additional water, the latter mechanism was more critical. The substantial reduction in autogenous shrinkage can be attributed to the internal curing effect provided by SAPs, which gradually release absorbed water, thereby maintaining internal relative humidity, homogenizing moisture distribution, and sustaining hydration during the early stages of curing. Figure 9 depicts the drying shrinkage trends of HPC mixtures subjected to three levels of extended mixing. Baseline measurements were taken after 7 days of curing in a lime-saturated solution. By 21 days, all mixtures showed shrinkage values below 300 µ strain. The addition of SAPs effectively suppressed shrinkage at all mixing durations. In particular, incorporating SAP-P lowered the 28-day drying shrinkage by 43–76%, depending on the mixing time.

The consistent performance observed across the three mixing durations also suggests that a 3 min extended mixing time is sufficient to achieve uniform dispersion of SAP particles throughout the matrix. Adequate dispersion is critical to maximize the effectiveness of SAPs, as it ensures a homogeneous distribution of curing water across the cementitious matrix. These findings have practical implications for field applications, as they highlight the potential to optimize mixing protocols without compromising the internal curing efficiency or shrinkage control. In particular, minimizing extended mixing time without sacrificing SAP dispersion may contribute to improved productivity and energy efficiency in ready-mix concrete production.

### 3.5. Transport Properties

Table 5 presents the electrical surface and bulk resistivity values of the HPC mixtures prepared with varying extended mixing times. The surface resistivity of SAP-modified mixtures ranged from 56 to 59 Ω·m across all three mixing durations, indicating good resistance to chloride ion penetration according to established classifications. In addition, the incorporation of SAPs led to a notable increase in bulk resistivity compared to the reference mixture. Specifically, the bulk resistivity of mixtures containing SAP-P ranged from 167 to 182 Ω·m, whereas the reference mixture exhibited a lower value of 147 Ω·m. This enhancement is likely attributed to the improved microstructure and refined pore system resulting from prolonged internal hydration facilitated by SAPs. Importantly, no significant differences in either surface or bulk resistivity were observed among the extended mixing times (3, 5, and 7 min), suggesting that SAP dispersion and its beneficial effects on resistivity were not sensitive to variations in mixing duration within the studied range.

Previous studies [46,47] have demonstrated that acrylamide/acrylic acid copolymer-based SAPs can improve the sealing performance and reduce water permeability of engineered cementitious composites, with the efficiency strongly influenced by particle size. Specifically, SAPs with a mean particle size of 550 μm were found to be more effective in filling cracks and mitigating water transport than those with smaller sizes (e.g., 75 μm). In the present study, a similar trend was observed: the mixtures incorporating SAP-P, which has a mean particle size of 850 μm, exhibited higher uniaxial electrical resistivity than those made with SAP-B (63 μm). This suggests that larger SAP particles may contribute more effectively to enhancing resistance to ionic penetration.

## 4. Conclusions

This study examined the influence of superabsorbent polymers (SAPs) with varying absorption kinetics and extended mixing times on the rheological, mechanical, shrinkage, and transport properties of high-performance concrete (HPC). Based on the experimental results, the following conclusions are drawn:

1. The incorporation of SAPs increased the superplasticizer (SP) demand and plastic viscosity due to continued moisture absorption during extended mixing. Yield stress remained relatively stable across mixing durations, indicating a compensatory effect between SAP absorption and SP dispersion.
2. SAPs significantly enhanced static yield stress and reduced surface settlement, thereby improving the fresh stability of HPC. Extended mixing time had minimal additional effect on fresh stability once adequate dispersion was achieved.
3. Mechanical performance, including compressive strength, flexural strength, and modulus of elasticity, was consistently improved by 10–25% with SAP addition. These enhancements were not sensitive to mixing duration, suggesting that a 3 min extended mixing period was sufficient to ensure effective SAP dispersion.
4. Both autogenous and drying shrinkage were markedly reduced with SAPs, confirming their effectiveness as internal curing agents. The reduction in shrinkage was largely independent of mixing time.
5. Electrical resistivity measurements showed improved durability performance in SAP-modified mixtures. No significant variation was observed across the tested mixing durations, further supporting the robustness of SAP benefits under practical mixing conditions.

## Figures and Tables

**Table 1:** Physical and chemical characteristics of cementitious materials.

Binder	SiO_2_ (%)	Al_2_O_3_ (%)	Fe_2_O_3_ (%)	CaO (%)	MgO (%)	SO_3_ (%)	Na_2_O eq., %	LOI (%)	Blaine Surface Area, m^2^/kg	Specific Gravity
OPC	19.0	3.9	3.5	68.3	1.7	2.4	0.6	1.5	390	3.14
FA	40.4	19.8	6.3	24.4	3.5	1.0	1.3	3.4	490	2.71
Slag	36.2	7.7	0.7	44.2	7.6	1.7	0.52	0	530	2.86

**Table 2:** Mixture proportion of investigated concrete.

No.	Materials (kg/m^3^)							Admixtures (wt.% of Binder)		
	Cement	Slag	FA	Sand	CA	Water	SAP	SP	AEA	Retarder
Control	184	125	47	736	1074	132	0	0.15	0.07	0.12
P-M3							0.9	0.25		
P-M5								0.28		
P-M7								0.34		
B-M3							0.7	0.26		
B-M5								0.26		
B-M7								0.28		

P and B denote SAP-P and SAP-B, respectively; M3, M5, and M7 denote 3 min, 5 min and 7 min of extended mixing times, respectively.

**Table 3:** Fresh properties of HPC made with SAPs and different extended mixing times.

Mixture	Slump (mm)			Air Volume (%)		Unit (kg/m^3^)	SP Content (wt.% of Binder)		
	Initial *	20 min	40 min	Initial *	40 min		Before SAP Addition	After SAP Addition	Total
Reference	216	198	178	5.6	6.4	2326	0.15	-	0.15
P-M3	218	147	66	5.1	5.6	2338		0.9	0.24
P-M5	218	127	53	5.5	5.6	2333		0.13	0.28
P-M7	224	169	136	5.8	6.5	2330		0.19	0.34
B-M3	218	183	160	5.2	5.5	2331		0.11	0.26
B-M5	208	173	137	5.5	6.0	2323		0.11	0.26
B-M7	213	178	157	5.7	6.4	2325		0.13	0.28

* The initial slump and air content were measured 2 min after cement–water contact for the reference concrete, and at 12, 14, and 16 min for the mixtures containing SAP with extended mixing times of 3, 5, and 7 min, respectively.

**Table 4:** Rheological properties of the HPC mixtures made with SAPs and different extended mixing times.

Mixture	Yield Stress (Pa)	Plastic Viscosity (Pa·s)	Static Yield Stress at 5 min (Pa)
Reference	76	79	310
P-M3	372	178	832
P-M5	356	234	799
P-M7	363	292	410
B-M3	330	142	598
B-M5	338	153	612
B-M7	345	158	643

**Table 5:** Electrical resistivity of HPC made with SAPs and different extended mixing times.

Mixture	Surface Resistivity (Ω·m)	Uniaxial Resistivity (Ω·m)
Reference	55.1	147.3
P-M3	58.6	173.0
P-M5	58.3	181.7
P-M7	57.5	167.1
B-M3	59.1	165.5
B-M5	56.8	165.9
B-M7	56.1	163.3

## Data Availability

The original contributions presented in this study are included in the article. Further inquiries can be directed to the corresponding author.
